# Insights into fungal communities in some benchmark agricultural soils of Alberta, Canada

**DOI:** 10.1099/mic.0.001704

**Published:** 2026-05-05

**Authors:** Nafsa Khazaei, M. Derek MacKenzie, Brian Lanoil

**Affiliations:** 1 Department of Biological Sciences, University of Alberta, Edmonton, AB, T6G2R3, Canada; 2 Department of Renewable Resources, University of Alberta, Edmonton, AB, T6G2R3, Canada

**Keywords:** agricultural parameters, community heterogeneity, functional traits, fungal diversity, ITS2 (internal transcribed spacer) region

## Abstract

Soil micro-organisms, including fungi, are integral to soil functions such as biogeochemical cycling, pollutant degradation and plant growth promotion. Soil fungi are influenced by environmental perturbations such as agricultural parameters and thus may be a potential soil health indicator. We hypothesized that tillage intensity, crop type and herbicide application would impose changes on soil fungal community composition and diversity and that these shifts would be associated with changes in soil physico-chemical properties. We examined the response of soil fungal communities to various agricultural parameters at a provincial scale from benchmark agricultural sites in Alberta. Surface soil samples from 26 farm locations were collected from benchmark sites across Alberta. Samples were grouped by crop type, tillage intensity and herbicide application. Physico-chemical properties of soil samples were measured. Following DNA extraction of soil samples, we used amplicon sequencing through primers for the ITS2 (internal transcribed spacer) region and analysed fungal community diversity, composition and predicted function. Multivariate analyses showed that ecoregion was the strongest environmental predictor of variation in fungal community composition. Amongst farming practices and soil properties, crop type was the most important identified driver of fungal community composition, followed by tillage intensity and soil pH. The genera *Mortierella*, *Pseudogymnoascus* and *Fusicolla*, as well as functional traits, including soil saprotrophs and mycoparasites, were responsive to variation in crop type, tillage levels and herbicide application. We observed minor changes in overall fungal diversity, but significant differences in species composition, in all farming treatments. The heterogeneity of samples also varied significantly by tillage intensity and crop type. No indicator species was strongly associated with crop type, tillage intensity or herbicide system. Bray–Curtis dissimilarity in the fungal community was positively correlated with soil pH and base saturation and negatively correlated with soil cation exchange capacity, organic matter and clay content. We conclude that management practices affect different attributes of soil fungal communities, and fungal community heterogeneity is a responsive attribute that could be used for soil health assessment in Alberta. The potential linkage we found between agricultural parameters, fungal community heterogeneity and soil parameters could be further investigated as a metric in soil health assessment.

## Graphical Abstract

Soil genomic data focusing on soil fungi can relate agricultural processes to soil health.

## Data Availability

The sequencing data used in this study were deposited in the National Center for Biotechnology Information of the National Library of Medicine under BioProject PRJNA1186712 with the accession numbers SRR31375431–SRR31375664. Other data, including soil chemistry, survey results and geolocation data, are available in the supplementary information.

## Introduction

Soils provide multiple ecosystem functions, including habitat for various organisms, nutrient cycling, water purification, carbon storage and food security [1]. In 2017, the United Nations reported a list of potential global soil threats, including erosion, organic carbon loss, nutrient imbalance of inputs and outputs, salinization, biodiversity loss, contamination and compaction [2]: all issues that potentially make soils ‘unhealthy’. Soil is a complex and heterogeneous ecosystem with various physico-chemical and biological interactions and processes [3]. Therefore, efforts to define and assess soil health have been controversial. The most agreed-upon definition is ‘soil health is the continued capacity of soil to function as a living ecosystem that sustains plants, animals, and humans’ [4]. Since soil health reflects the physico-chemical and biological composition of soil [5], biotic and abiotic factors structuring soil properties are indicators of soil health.

Micro-organisms are key soil inhabitants that drive multiple soil functions [6]. Fungi and bacteria, with various functionalities and trophic modes, are the most studied soil microbial groups. Fungi constitute a diverse microbial community in soil with a strong ability to adapt to environmental disturbances and are able to survive in a wide range of soil pH and temperature [7,8]. Fungi play key ecological roles in soil ecosystems. Saprotrophic fungi decompose complex soil organic matter and dead material, regulating soil organic matter turnover and impacting soil carbon stabilization [9,10]. Filamentous fungi increase soil aggregate stability and thereby protect soil structure [11]. Symbiotrophs interact positively with their hosts, for example, mycorrhizal fungi interact with plant roots, increasing the rate of nutrient uptake from soil [12]. Pathotrophic fungi negatively interact with their hosts; for example, mycoparasitic soil fungi kill other fungi, which could be harmful to plants by inhibiting symbiotrophic fungal partners [13] or beneficial by protecting against plant pathogenic fungi [14]. These roles of soil fungi highlight their importance in determining soil health, and the diversity of fungal communities is a key determining factor in ecosystem functioning and sustainability [15]. Community genome analysis of soil becomes useful in soil health assessment when information on microbial taxonomic and functional diversity can be linked to soil ecosystem functions.

Shifts in environmental conditions, as well as agricultural parameters such as application of soil fertilizer and tillage intensity, can impact soil fungal communities, which in turn can potentially alter soil functioning. Application of nitrogen fertilizer in combination with soil amendments, such as biochar, changes soil physico-chemical properties such as pH, resulting in alteration of interactions between fungal communities and the abundance and diversity of important fungal groups that drive the degradation of organic compounds and nutrient cycling [16]. High tillage intensity is associated with reductions in soil organic matter and nutrient concentrations, which affect the abundance of fungal genera associated with symbiotrophic, saprotrophic and pathotrophic functional modes [17]. Application of different crop types also leads to changes in fungal community diversity and structure, which could affect soil nutrient dynamics and ecosystem functioning [18,19].

Soil fungi tend to respond differently to agricultural parameters at different locations. For example, in a study of 12 long-term experiments and 20 agricultural treatments across Europe, Hannula *et al.* [20] found that fungal communities vary by geographic location, and the directionality of fungal community response to agricultural parameters is not consistent from location to location but rather responds to soil abiotic conditions, crop type and climatic conditions [20]. In a study of the fungal composition and diversity of soils, Tedersoo *et al.* [21] found that climatic factors, which are correlated with geographic region, followed by edaphic and spatial patterning, are the best predictors of soil fungal parameters at the global scale [21]. Bahram *et al.* [22] found that bacteria and fungi had contrasting responses to environmental variables and appeared to be antagonistic towards each other at a global scale [22], indicating that bacterial and fungal communities vary spatially and are controlled by both competition and environmental filtering. In addition, Labouyrie *et al.* [23] found that the spatial distribution of soil fungi and bacteria across Europe is best explained by interactions between vegetation, climate and soil properties [23]. As a result, it seems likely that fungal communities vary at regional, continental and global scales, and their responsiveness and the directionality of response to agricultural parameters are site dependent.

From 1997 to 2006, the Soil Quality Monitoring Project (SQMP) was established to examine baseline metrics and the impacts of agricultural management practices on soil quality at benchmark sites across Alberta. In 2019–2020, we resampled these sites with the goal of expanding the findings from the original SQMP to include soil microbial community metrics. The initial study of bacteria at the resampled sites indicated that agricultural treatments did not affect bacterial community diversity or composition but did affect bacterial community interactions as assessed by co-occurrence networks [24]. However, other studies indicate that while bacteria are primarily controlled by soil edaphic and climate parameters, fungi are more correlated with land management intensity [25], and soil fungi respond more strongly to agricultural treatments compared to bacteria [26]. Therefore, we aimed to study the fungal communities of surface soils from multiple ecoregions as defined by Cathcart *et al.* [27], described by soil type, climate and vegetation mode at the provincial scale, which varied in crop type, tillage intensity and herbicide usage. We hypothesized that differences in these farming practices impose changes on soil fungal community composition and diversity, and these shifts are associated with changes in soil physico-chemical properties. In our study, agricultural soil samples belonged to various geographic locations, and due to soil heterogeneity across Alberta, we hypothesized that ecoregion and soil type affect soil fungal communities more strongly than agricultural parameters.

To our knowledge, relatively few studies have reported the responses of fungal communities in agricultural soils at large scales in North America. We were interested in evaluating if the trends that previous studies found at global or continental scales in soil fungal communities’ response to agricultural parameters can be found across the province of Alberta. Also, due to the lack of any universally approved microbial genomic indicator for soil health assessment [28], we aimed to evaluate the potential application of fungal genomic data as a soil health indicator in Alberta.

## Methods

### Site description, sampling and physico-chemical analysis

Soils were collected from the original SQMP locations in 2019 and 2020 (Fig. S1, available in the online Supplementary Material [27]). For microbial analysis, three 5-cm-diameter replicate soil samples, from 0 to 15 cm in depth using a soil core extraction probe, were collected from each of three slope positions along a catena: upper, middle and lower. For physico-chemical analyses, composite samples were collected as a mixture of ten core samples in a 3 m radius from a central point at each slope position, for a total of three composite samples per site. Soil samples were collected in bags and stored on ice in cooler boxes until transferred to the laboratory. In the laboratory, soils were stored at −80 °C for a year prior to further analysis. Physico-chemical properties, including soil texture, bulk density (BD), nutrients, base saturation (BS), cation exchange capacity (CEC), total carbon and nitrogen, organic matter content (OM), pH and electrical conductivity (EC), were measured at Element Material Technology (Edmonton, AB, Canada) using standard methods (Table S1 [29]).

Information regarding specific farming practices, including tillage intensity, crop types and herbicide used, was obtained from an online survey (Table S2) and was based on producers' self-reported land usage for the year of collection. Based on the availability of farmers’ surveys, we examined soil samples from 26 distinct farms across Alberta (Fig. S1, Table S2). Table S3 describes samples based on their farming practices. Samples were grouped by tillage intensity, fertilization method, crop type and herbicide usage. Samples were categorized by producer-reported tillage intensity into ‘zero’ (no passes), ‘low’ (one pass) or ‘high’ (two or more passes). Crop types included alfalfa, barley, canola (a.k.a. oilseed rape), durum, fallow, forage, hay, livestock, sugar beets, wheat and none. Crops under the ‘none’ category consisted only of abandoned sites. Crops, including alfalfa, hay, sugar beets and no crop, had only one representative farm each, so we therefore excluded those sites from the pairwise analysis of the effects of crop variation on soil fungal community, leaving 22 farms for crop type analysis (Table S3). Considering other crop types, wheat, durum and barley were all cereal crops and were included in a single group named ‘grains’; fallow, forage and livestock farms shared common characteristics related to long-term ground cover and were included in a single group named ‘perennial’; and canola was considered a single group. All sites receiving herbicide treatments were grouped together, regardless of the herbicide type, and were compared to sites receiving no herbicide application (Table S2). Due to the lack of farm replicates within each ecoregion/geographical location (terms will be used interchangeably), to assess the effect of agricultural parameters on fungal community composition and diversity, we considered all farms across all ecoregions together.

### DNA extraction and internal transcribed spacer sequencing

For microbial analyses, roots were removed by hand, and soils were homogenized by passage through a 4 mm sieve and frozen at −80 °C until DNA extraction was performed. DNA was extracted from a duplicate 0.25 g subsample of the sieved soil using the DNeasy PowerSoil Pro^®^ kit (QIAGEN, Germany). Libraries of PCR products were produced from the extracted DNA by Microbiome Insights (Vancouver, BC, Canada) using a 300 bp paired-end kit (Illumina MiSeq v3 kit) using ITS2 (internal transcribed spacer) forward and reverse primers [30] targeting the ITS2 region of fungal DNA [31].

### Bioinformatics and statistical analysis

Demultiplexed raw reads from Illumina sequencing were used as input for the DADA2 v1.8 pipeline [32] in RStudio v4.2.1 [33]. Reads were processed in a stepwise workflow using default parameters in the ITS-specific DADA2 pipeline [32]. In total, 60% of reads remained after quality control, merging paired-end reads and chimaera removal.

Amplicon sequence variants (ASVs) consisted of inferred unique sequences from the core sample inference algorithm in DADA2 v1.8 clustered at 100% similarity and were considered analogous to fungal species [32], producing 12,609 ASVs from 1,878,395 reads. We used the *decontam* package v1.1 [34] in RStudio; 31 ASVs were identified as potential contaminants and removed. Library sizes were normalized by multiple rarefaction to the smallest library size, ~5,000 reads in *vegan* package v2.6 [35]. Taxonomy was assigned to ASVs using the UNITE database [36] with the *assignTaxonomy* function in the DADA2 pipeline. A total of 70% and 45% of ASVs were classified at the phylum and genus levels, respectively. We extracted unique fungal genera using the *phyloseq* package v1.47 [37] with the *tax_glom* function. We manually annotated fungal genera to their corresponding traits and growth forms using the FungalTraits database [38].

To determine the effect sizes of agricultural treatments and soil physico-chemical properties, we used two different approaches: permutational multivariate analysis of variance (PERMANOVA) using the *adonis2* function and multiple regression with redundancy analysis (RDA) using the *rda* function, both in the *vegan* R package [39]. To assess the effect of agricultural parameters on soil properties, fungal diversity and functional and taxonomic composition, the homogeneity of variance was tested for groups of farming practices using Levene’s test as implemented in the *car* package [40]. If treatments showed homogenous variance, we used ANOVA and Tukey HSD set to a confidence level of 0.95 to test for significant differences in pairwise comparisons using the base packages in RStudio. If treatments showed heterogeneous variance, we used the Kruskal–Wallis test (in base R package) and Dunn’s test, as implemented in the *FSA* R package [41], to test for significant differences in pairwise comparisons. Fungal alpha diversity metrics, including observed ASV richness, Chao1 non-parametric richness index, Shannon–Wiener diversity index and inverse Simpson diversity index, were calculated using the *estimate_richness* function in the *phyloseq* package and compared in a pairwise manner between treatments. We used non-metric multidimensional scaling (NMDS) with Bray–Curtis dissimilarity using the *phyloseq::distance* function in the *phyloseq* package to visualize differences in fungal species composition between samples. Based on observations from the NMDS, we used the *pairwise.adonis* function in the *Adonis* package to test the hypothesis of significant differences in species composition between groups of farming practices. We calculated Bray–Curtis dissimilarity between samples using the *vegdist* function in the *vegan* R package and performed pairwise tests of the significance of the differences in mean heterogeneity between each farming practice group through Tukey HSD or Dunn’s test, based on the heterogeneity of variance mentioned above. We calculated the strength and direction of the association between soil parameters and fungal composition and diversity using the Spearman correlation coefficient using the *rcorr* function in the ‘*Hmisc*’ package v5.1–0 [42]. For all tests, we used *P*<0.05 as the cutoff for statistical significance, otherwise indicated.

## Results

In this study, we revisited 26 farms across the Canadian province of Alberta that were originally established in SQMP [27], and we assessed soil physico-chemical properties, producer-reported agricultural parameters (from a farmer survey) and soil fungal community diversity and composition. Samples were grouped based on agricultural parameters into different tillage levels (‘zero’, ‘low’ and ‘high’), different crop types (‘grains’, ‘perennial’ and canola) and herbicide application (‘herbicide’, ‘no herbicide’). With the taxonomic assignment of fungal ASVs, we used the FungalTraits database to assign each fungal genus to their corresponding functional trait (examples in Table S4).

### Fungal community analysis

There were no significant differences in fungal within-sample diversity (i.e. alpha diversity, measured by taxon richness, evenness or Shannon–Wiener diversity index) or species composition based on slope position (Fig. S2). Therefore, we treated the samples from the three slope positions as farm replicates.

Ecoregion was the feature that had the largest effect on variation in fungal community composition, although with different effect sizes in the two statistical analyses (21% in PERMANOVA analysis and 13.6% in RDA analysis; Table 1). Amongst agricultural parameters, the most variation in fungal community composition was explained by crop type (12%) and tillage intensity (7%), and amongst soil properties by soil pH (6%) (Table 1). All other measured environmental factors were significantly related to variation in fungal community composition, but their effect sizes were small (all less than 4%). RDA analysis showed a total variation of 66% in fungal community composition, of which ~45% was explained by the included factors and ~55% was either explained by environmental factors that were not included in our study or by stochastic processes. Similar to the values of PERMANOVA analysis, crop type (8%), tillage levels (3.1%) and soil pH (0.8%) were significant drivers of variation in fungal community composition based on RDA findings; however, unlike the PERMANOVA values, other measured factors were not significantly correlated with changes in fungal community composition (Table 1).

**Table 1.** Contribution of explanatory environmental factors on responses of fungal and bacterial community variance. *** (*P*-value<0.001), ** (*P*-value<0.01) and * (*P*-value<0.05)

Variable	Explanatory (%)			
	Fungi		Bacteria	
	PERMANOVA	RDA	PERMANOVA	RDA
Ecoregion	21.0***	13.6***	9.8*	10.7***
Crop	12.2***	8.0***	7.0	5.4***
Tillage	7.2***	3.1***	3.1	2.6***
Herbicide	2.6***	1.1**	1.7	1.3**
pH	6.2***	0.8*	1.8	0.8
BS (%)	3.8***	0.6	1.3	0.6
Clay (%)	3.7***	0.6	1.1	0.6
BD (kg/l)	3.6***	0.7	1.6	0.6
CEC (meq/100 g)	3.5***	0.5	1.6	0.5
OM (%)	3.2***	0.4	2.3*	0.6
C:N (%)	3.0***	0.7	2.8**	0.9
NH_4_ (mg/kg)	2.6***	0.7	1.3	1.0
NO_3_ (ppm)	2.5**	0.6	1.1	0.7
EC (dS/m)	2.4**	0.5	1.4	0.6
PO_4_ (ppm)	2.1*	0.7	1.4	0.8
Silt (%)	2.1**	0.6	0.8	0.6

### Influence of crop type on fungal communities

In canola, perennial and grain samples, soil fungal communities were dominated by the fungal genera *Mortierella* and *Pseudogymnoascus* (Table 2). Both of these genera have filamentous growth styles and saprotrophic traits in soils that degrade organic matter [38]. Amongst the ten most abundant genera in our samples, only two showed significantly different abundance by crop type: *Mortierella* had higher relative abundance in canola soils, and *Fusicolla* had higher relative abundance in grain soil sites relative to the other crops. Soil saprotrophs were the most abundant fungal trait in all crop groups. Interestingly, different crop types were not significantly associated with fungal functional composition (Table 2).

**Table 2.** Relative abundance of the most abundant fungal genera and traits with crop types, tillage levels and herbicide usage. Compact letters show significant differences

**Agricultural parameters**		**Crop type**			**Tillage intensity**			**Herbicide application**	
**Genus**		**Canola**	**Perennial**	**Grains**	**High**	**Low**	**Zero**	**Herbicide**	**No herbicide**
*Mortierella*		0.22^a^	0.13^ab^	0.14^b^	0.13^a^	0.17^a^	0.16^a^	0.15^a^	0.18^a^
*Pseudogymnoascus*		0.13^a^	0.19^a^	0.15^a^	0.09^a^	0.19^b^	0.14^ab^	0.14^a^	0.2^b^
*Fusicolla*		0.07^ab^	0.03^a^	0.10^b^	0.18^a^	0.07^b^	0.05^b^	0.09^a^	0.04^b^
*Solicoccozyma*		0.03^a^	0.03^a^	0.05^a^	0.07^a^	0.03^b^	0.04^ab^	0.05^a^	0.03^a^
*Tetracladium*		0.03^a^	0.05^a^	0.04^a^	0.03^a^	0.05^a^	0.03^a^	0.03^a^	0.05^a^
*Humicola*		0.06^a^	0.03^a^	0.02^a^	0.02^a^	0.02^a^	0.04^a^	0.03^a^	0.02^a^
*Vishniacozyma*		0.02^a^	0.01^a^	0.02^a^	0.02^a^	0.02^a^	0.02^a^	0.02^a^	0.02^a^
*Coniochaeta*		0.01^a^	0.01^a^	0.02^a^	0.03^a^	0.02^a^	0.02^a^	0.02^a^	0.01^a^
*Leptosphaeria*		0.01^a^	0.03^a^	0.02^a^	0.01^a^	0.02^a^	0.01^a^	0.01^a^	0.02^a^
*Tausonia*		0.003^a^	0.005^a^	0.01^a^	0.01^a^	0.02^a^	0.01^a^	0.02^a^	0.004^a^
**Broad functional traits**	**Substrate**	**Canola**	**Perennial**	**Grains**	**High**	**Low**	**Zero**	**Herbicide**	**No herbicide**
Soil saprotroph	Root, soil	0.4^a^	0.4^a^	0.4^a^	0.4^a^	0.5^b^	0.4^ab^	0.4^a^	0.5^a^
Litter saprotroph	Leaf, seed	0.08^a^	0.1^a^	0.1^a^	0.08^a^	0.1^a^	0.1^a^	0.08^a^	0.1^b^
Mycoparasite	Fungal material	0.09^a^	0.05^a^	0.1^a^	0.2^a^	0.08^b^	0.07^b^	0.1^a^	0.07^a^
Wood saprotroph	Wood, leaf, seed	0.1^a^	0.06^a^	0.07^a^	0.07^a^	0.05^a^	0.08^a^	0.07^a^	0.05^a^
Unspecified saprotroph	–	0.05^a^	0.07^a^	0.06^a^	0.06^a^	0.06^a^	0.06^a^	0.06^a^	0.06^a^
Dung saprotroph	Dung, animal material	0.02^a^	0.04^a^	0.04^a^	0.02^a^	0.05^a^	0.03^a^	0.04^a^	0.04^a^
Animal parasite	Animal material	0.03^a^	0.02^a^	0.03^a^	0.03^a^	0.02^a^	0.03^a^	0.03^a^	0.03^a^

**Fig. 1.**
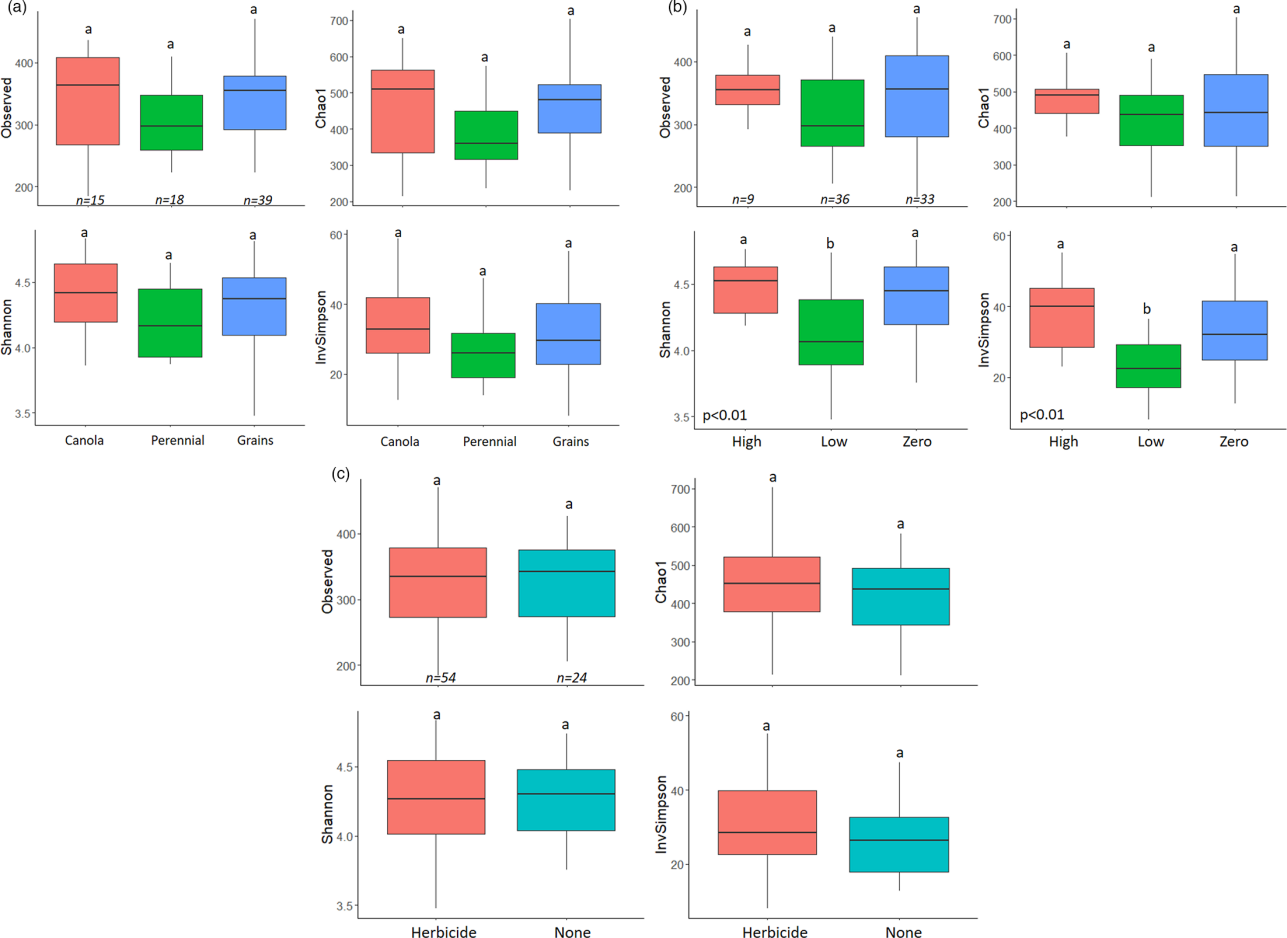
Alpha diversity metrics [Observed (Richness), Chao1, Shannon and InvSimpson] of fungal communities by different crop types (**a**), tillage levels (**b**) and herbicide usage (**c**). Letters show statistically significant groups.

**Fig. 2.**
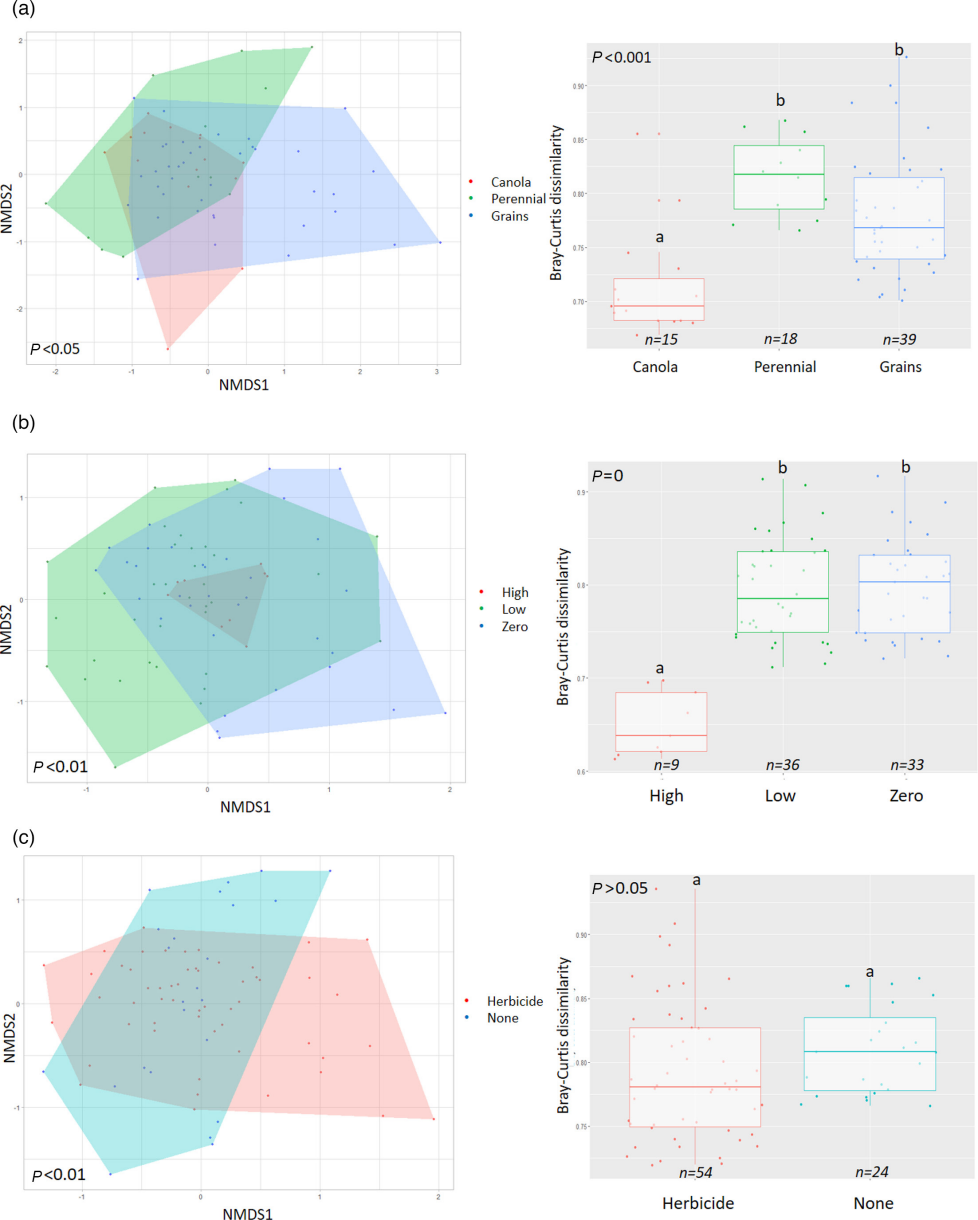
Bray–Curtis dissimilarity of fungal communities by different crop types (**a**), tillage levels (**b**) and herbicide usage (**c**). Letters show statistically significant groups. Left figures show species composition (NMDS), and right figures show pairwise Bray–Curtis dissimilarity, a measure of community heterogeneity.

Fungal alpha diversity metrics, including observed ASV richness, estimated ASV richness (Chao1), the Shannon–Wiener diversity index and the InvSimpson index, did not vary significantly between canola, perennial and grain samples (Fig. 1). Fungal community composition and heterogeneity significantly changed by crop type (Fig. 2). Fungal communities in perennial and grain samples were more dissimilar within the group than in canola, possibly indicating that canola planting led to convergence of fungal community composition (Fig. 2). However, in indicator species analysis, we found no strong indicator species with more than an 80% chance of appearance for any of canola, perennial and grain crops, indicating that there were no ASVs strongly associated with any specific crop types (Table S5).

### Influence of tillage levels on fungal communities

The fungal community for high-, low- and no-till soils was dominated by the genera *Fusicolla*, *Pseudogymnoascus* and *Mortierella*, respectively (Table 2). Low-till samples had a higher relative abundance of *Pseudogymnoascus* and a lower relative abundance of *Solicoccozyma* compared with high-till samples. *Fusicolla* was more abundant in high-till samples than in low-till or no-till samples. Soil saprotrophs were significantly more abundant in low-tillage soils relative to high-tillage soils, while mycoparasites were at higher abundance in high-tillage soils compared with low- and no-tillage soils (Table 2). High- and no-till soils had higher diversity metrics (i.e. Shannon and InvSimpson indices) than low-till soils (Fig. 1). High-till soil fungal communities were significantly less heterogeneous compared with low- or no-till ones (Fig. 2). However, in indicator species analysis, we found no indicator species (with more than 80% chance of appearance) associated with any of the tillage levels, indicating that there were no specific ASVs strongly associated with any of the tillage levels (Table S5).

### Influence of herbicide usage on fungal communities

The dominant fungal genus in soils with herbicide application was *Mortierella*, whereas soils without herbicide application were dominated by the fungal genus *Pseudogymnoascus* (Table 2). Litter saprotrophs were more abundant when no herbicide was applied to soil, but other functional groups did not differ based on herbicide usage (Table 2). Fungal alpha diversity metrics did not differ significantly between herbicide and no herbicide application (Fig. 1). The species composition varied significantly between herbicide and no herbicide usage; however, the community heterogeneity did not differ significantly between herbicide- and non-herbicide-treated soils (Fig. 2). However, in indicator species analysis, we found that no strong indicator species was associated with any of the herbicide application groups, indicating that herbicide utilization did not significantly affect the presence/absence or abundance of any specific fungal ASVs (Table S5).

### Correlations of agricultural parameters and fungal communities with soil edaphic parameters

Some soil edaphic parameters differed significantly in different agricultural treatments. pH was higher in low-tillage and no-herbicide samples; BS was higher in no-herbicide samples; and OM was higher in perennial and no-herbicide samples (Table 3). However, the majority of soil edaphic parameters did not differ significantly in soils from different agricultural parameters (Table 3). Thus, only a few indicative soil physico-chemical properties showed weak and inconsistent correlation with agricultural treatments.

**Table 3.** Statistical comparison of means of soil physico-chemical properties within crop type, tillage levels and herbicide usage. Bold shows higher values. *** (*P*-value<0.001), ** (*P*-value<0.01) and * (*P*-value<0.05)

Variables	pH	BS	Clay	BD	CEC	OM	C:N	NH_4_	NO_3_	EC	PO_4_	Silt
**Crop**	ns†	ns	ns	ns	ns	Perennial-wheat*	ns	ns	ns	ns	ns	ns
**Tillage**	Low-high*	ns	ns	ns	ns	ns	ns	ns	ns	ns	ns	ns
**Herbicide**	No herbicide*	No herbicide*	ns	ns	ns	Herbicide*	ns	ns	ns	ns	ns	ns

†ns: not significant.

**Fig. 3.**
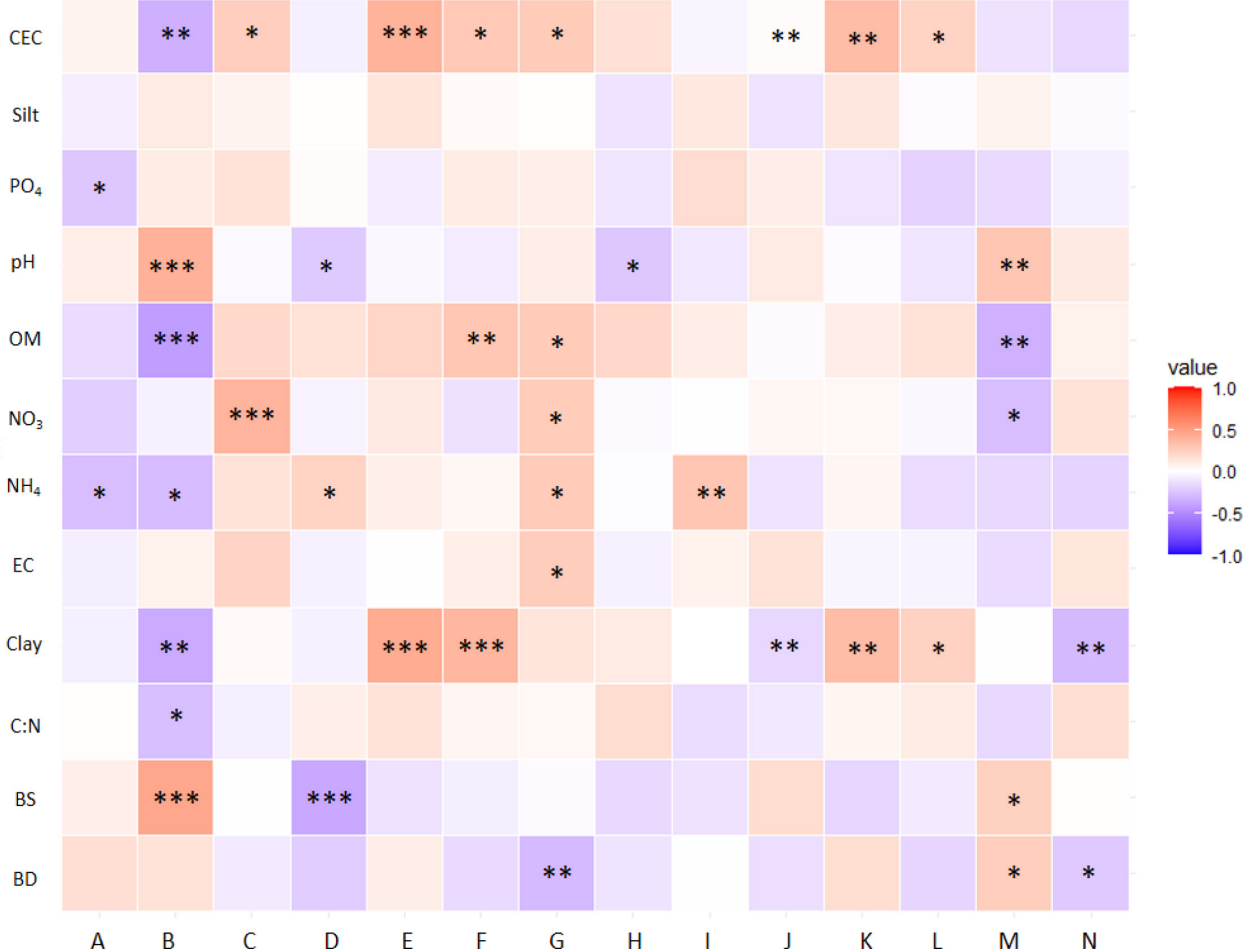
Heat map showing the Spearman correlation coefficient of soil parameters with fungal diversity, abundant genera and functional composition. *** (*P*-value<0.001), ** (*P*-value<0.01) and * (*P*-value<0.05). Shannon diversity index (**a**), Bray–Curtis dissimilarity (**b**), relative abundance of *Mortierella* (**c**), relative abundance of *Pseudogymnoascus* (**d**)*,* relative abundance of *Fusicolla* (**e**), relative abundance of *Solicoccozyma* (**f**), relative abundance of *Tetracladium* (**g**), relative abundance of *Humicola* (**h**), relative abundance of soil saprotroph functional group (**i**), relative abundance of litter saprotroph functional group (**j**), relative abundance of mycoparasite functional group (**k**), relative abundance of wood saprotroph functional group (**l**), relative abundance of unspecified saprotroph functional group (**m**) and relative abundance of dung saprotroph functional group (**n**).

We found few significant correlations between the Shannon–Wiener alpha diversity metrics and soil edaphic parameters, with only inorganic nutrient concentrations (PO_4_ and NH_4_) correlating (Fig. 3). The Bray–Curtis dissimilarity metric, a measure of variability within an agricultural treatment, correlated with more edaphic parameters, with >50% of edaphic parameters correlating and showing fairly strong correlation values with pH, OM and BS (r>0.4) (Fig. 3). Thus, overall fungal diversity appeared to be less correlated with edaphic parameters than fungal community composition heterogeneity.

We examined the correlation between edaphic parameters and community composition further by examining the relative abundance of highly abundant fungal genera. For a genus to be included in the analysis of correlations with soil properties, we set a threshold of 5% relative abundance in at least one category of farming practices. Several genera were highly correlated with edaphic parameters. In particular, the relative abundance of genus *Tetracladium* was correlated with 50% of the edaphic parameters, although the correlation values were relatively weak (r<0.3). The strongest correlations were seen between the relative abundance of genus *Fusicolla* and CEC and clay content, the genus *Pseudogymnoascus* and BS and the genus *Solicoccozyma* and clay content. Thus, some highly abundant genera appeared to be correlated with specific edaphic parameters.

Then, we examined the correlations between edaphic parameters and the functional composition of the fungal community. Similar to our genus correlation analysis, we limited our function trait correlation analysis to those functions with 5% or greater relative abundance in at least one agricultural treatment. As a result, most of the correlating fungal functions were related to a saprophytic lifestyle, with mycoparasitic function as the only exception (Fig. 3). The various saprophytic functions correlated with most of the edaphic parameters to various degrees. The most highly correlated saprotrophic group was the unspecified saprotrophic trait, which was most closely correlated with OM (r>0.4). Mycoparasites were only correlated with CEC and clay.

Taken together, these findings indicate that Bray–Curtis dissimilarity, the relative abundance of genus *Tetracladium* and saprophytic functional groups were the fungal community attributes most correlated with soil edaphic parameters. However, there were no edaphic parameters that correlated with all fungal community attributes.

We found correlations between (1) high pH and high community heterogeneity in low-till soils, (2) high BS and high community heterogeneity in no-herbicide application and (3) high OM and low community heterogeneity in perennial samples and herbicide application. These results support the hypothesis of a potential linkage between agricultural parameters, fungal community heterogeneity and soil parameters in this provincial-scale experiment.

### Responsiveness of bacterial communities to environmental variables

To directly compare the fungal community distribution in this study to the bacterial community distribution, we re-analysed the data from the previous bacterial study [24] as shown in Fig. S3, regrouping the samples to match our groupings and re-analyzing the edaphic parameters that we included here (i.e. three crop types and two herbicide categories; the three tillage groups remained the same as in the previous study). Specifically, we re-analysed the effect size of environmental and agricultural parameters on total variation in bacterial community composition (Table 1). Similar to the fungal community analysis (Table 1), ecoregion was the best explanatory variable in both PERMANOVA (9.8% of variance) and RDA (11% of variance) analyses. In terms of agricultural parameters, crop type, tillage intensity and herbicide application were significant in the RDA analysis, but not in the PERMANOVA analysis of bacteria (Table 1). Crop type and tillage parameters were ranked similarly as explanatory variables in both analyses, despite the lack of significance in the PERMANOVA analysis. In PERMANOVA values, C:N, OM and pH were better explanatory variables than herbicide, with C:N and OM showing significant correlation. In the fungal communities, the effect sizes of all three agricultural parameters were significant (Table 1). Thus, fungal and bacterial communities responded similarly to ecoregion and agricultural parameters, although the effect appeared to be larger and had stronger statistical support in the fungal communities.

## Discussion

We found that geographic location, which is directly related to both the soil type and climate of sampling sites, was the main environmental factor correlated with variation in fungal community composition. Our results supported previous findings that soil fungal community composition and diversity are primarily driven by climate and local edaphic parameters. A global meta-study indicates a large effect size of climate, temperature, precipitation and soil properties on fungal community distribution [43]. Another global analysis of fungal diversity shows a direct strong effect of climate, which indirectly affects soil edaphic parameters [21]. Findings from our previous SQMP study also showed that ecoregion was the main determinant of variance in bacterial communities [24]. Thus, the effects of agricultural parameters are not universal but appear to be dependent upon soil type and climate. The geographic location of sampling sites appears to be more influential on soil fungi than land management practices. It is important to note that due to the global heterogeneity of soils, regional-scaled studies are needed, and it appears that agricultural management practices do not have universally applicable impacts on soil microbes.

Most agricultural parameters and soil edaphic parameters had only small effects on total variation in fungal communities; however, some parameters had a significant influence on community structure (Table 1). Similar to our findings for the fungal community, based on our results from re-analyzing bacterial communities, agricultural and soil parameters corresponded to a small total variation in bacterial community composition, although most factors did not significantly influence bacterial community composition (Table 1). These findings contrast with previous studies that report major changes in fungal community composition and diversity as a result of variation in agricultural treatments such as crop rotation and tillage intensity [44,45]. In our study, we only had a single time point for agronomic and soil edaphic data, and benchmark sites were located on existing farms with a combination of different practices, which were grouped based solely on farmers’ survey data. Therefore, the history of farming practices and duration of their application is unknown. We hypothesize that there may be other factors that were not included in our study that may have driven variation in fungal communities, including (1) biological parameters such as interaction between fungal and bacterial communities or other soil components (e.g. mesofauna), the composition of the rhizosphere community (as opposed to the bulk soil community) or the rate of photosynthesis by plants and production of exudates; (2) chemical factors such as organic matter composition or the bioavailability of nutrients to soil fungi; (3) physical parameters such as soil compaction and porosity, soil temperature at the time of sampling, temporal changes or water-holding capacity; and/or (4) agricultural parameters such as crop rotation, seeding methods and amount of tillage in previous years. Since our experiment was uncontrolled and was conducted on existing farms across the province (living lab experiment), these findings should be further examined in experiments of agricultural parameters in more controlled settings.

When considering agricultural treatments, crop type was the strongest correlation to variation in fungal community composition. Our findings about the fungal communities were aligned with the results of our reanalysis of the bacterial community from the previous SQMP study (Table 1). Soil fungal communities shift over time in response to crop rotation [46] and perennial monoculture [47]. However, we compared changes in fungal communities between three categories of crop types across separate farms and seven ecoregions at one sampling time. Given that we measured the abundance of genera and functional groups at a single point in time, our data were not comparable with previous studies of crop management (such as cover crop, mono crop and crop rotation) effects on fungal community composition that occur over time. Canola, perennial and grain samples were selected from different ecoregions and diverse farming practices across Alberta. There are clear associations between plant and fungal communities [48]; thus, it was surprising that we did not observe a significant correlation between crop type, richness/evenness of fungal communities and soil edaphic parameters (except for OM, Table 3). In this study, agronomic performance and soil edaphic properties were assessed at a single sampling time point, which restricts our ability to capture temporal variability in soil conditions and management responses. In addition, benchmark sites were established on working farms where multiple management practices co-occurred. Consequently, management classifications relied on farmer-reported survey data, which may not fully capture the complexity or historical variability of on-farm practices. We used the ITS2 region to characterize soil fungal communities; however, this approach has inherent limitations, as some fungal taxa may have been underrepresented or missed due to primer specificity, and PCR-based amplification can introduce biases that influence community composition and relative abundance estimates. Nevertheless, samples were handled and processed identically, so we believe that any bias should be systematic (i.e. should apply equivalently across all samples); as a result, despite these constraints inherent to a living lab experiment, we believe that our broad findings are consistent with field conditions, and this study yielded meaningful and informative patterns.

The high proportion of *Mortierella* and *Pseudogymnoascus* in all our samples is consistent with the higher abundance of these groups in agricultural soils [49,50]. Our result indicated that the relative abundance of *Mortierella* and *Fusicolla* varied with crop type, which is supported by previous reports that studied the changes in population of these genera with crop rotation systems [51,54].

Interestingly, crop variation had no effect on the composition of fungal functional groups. However, previous studies indicate a direct effect of crop type on the abundance of fungal functional groups such as arbuscular mycorrhizal fungi [17,55]. We assume that the lack of variation in fungal functional groups is likely due to the relatively minor changes in genus composition; functional redundancy likely contributes to the stability of fungal functional traits [56].

Our previous bacterial study showed the highest bacterial diversity metrics in fallow samples [24]. Previous reports also indicate an increase in soil microbial diversity following livestock grazing [57,58]. In contrast, we did not observe differences in fungal diversity in perennial samples (which included fallow, livestock-grazed and forage samples) compared with canola and grain samples. In our study, soils with the least heterogeneous fungal community were seeded with canola, as a single crop. We assume that different crop types in the combined group of samples (livestock, hay, forage and fallow grouped as perennial and barley, durum and wheat grouped as wheat) (Tables S2 and S3) led to increased heterogeneity in fungal communities of the soil.

In our samples, physical soil disturbance (high tillage) made the soil fungal communities less heterogeneous (Fig. 2b), presumably due to homogenization of the soils. The abundance of the fungal genus *Pseudogymnoascus* changed with differing levels of tillage, with higher abundance in no and low tillage. Members of this genus have a filamentous growth style. Soil physical disruption could negatively affect fungal hyphal integrity and break down the networks between plant roots and fungi [59]. Conversely, in no-till management, stable soil aggregation improves fungal hyphae development [60,61]. In contrast, members of the genus *Fusicolla* are mycoparasites. Mycoparasites were found in higher abundance in high-till soils; this was the only fungal trait with noticeable differential abundance between tillage systems. Mycoparasitism is the antagonistic trait of fungi that produce secondary metabolites that kill or inhibit the growth of other fungal cells [62]. In conventional farming practices, including high tillage, there could be space limitation for soil fungal colonization due to physical disruption of the soil and, as a result, an increase in competition and antagonistic traits against others [63].

Low tillage decreased fungal alpha diversity metrics in Alberta agricultural soils (Fig. 1b). Interestingly, both conventional tillage (high tillage) and conservation tillage (no tillage) supported higher fungal alpha diversity metrics than low tillage. Degrune *et al.* [64] also find higher bacterial and fungal diversity in conventional tillage systems [64]. We speculate that the disturbance made by high tillage can create new niches for fungi and therefore increase fungal diversity. However, other studies indicate decreased microbial diversity associated with high tillage [65,67]. Similar to bacterial communities [24], high tillage increased the similarity of fungal community structure (Fig. 2b). In contrast, low- and no-till systems increased dissimilarity and between-sample diversity in fungal communities.

The effects of herbicide on soil biology are not consistent across studies. Depending on the dosage and the chemistry of the herbicide component, they can have marginal or short-term effects on soil micro-organisms [68]. We did not observe any significant changes in fungal diversity and community heterogeneity by herbicide application, which matches our previous SQMP study showing no significant effects of herbicides on the bacterial community [24] or our re-analysis of bacterial communities from regrouping samples (Table 1). We assume that at our farms, the rate and type of herbicide application did not largely affect microbial community structure and diversity.

In other studies, farming practices such as crop system and tillage intensity strongly affect soil edaphic parameters [69,70]. Thus, it is unclear why we do not see strong effects of agricultural treatments on soil parameters, which responded only weakly and inconsistently in our study (Table 3). We hypothesize that regional effects on soil edaphic parameters are stronger than those of land management practices. To test this hypothesis, we would need more extensive soil sampling, with a better understanding of the details of the agricultural parameters than we were able to obtain from our survey. Alternatively, the soil parameters could be affected by agricultural parameters over multiple years rather than the single year of data that we obtained from our producer survey. A more thorough history of agricultural parameters over multiple years may reveal stronger correlations with soil edaphic parameters.

There was no soil physico-chemical parameter that consistently affected all fungal community attributes (diversity, genus and functional trait abundance). A strong correlation between soil pH and the taxonomic composition of soil fungal communities has been previously reported [21]; however, we only found a strong correlation between pH and the heterogeneity of fungal communities. Compared to other soil parameters, CEC and clay content were correlated with more fungal community attributes, including Bray–Curtis dissimilarity and taxonomic composition (Fig. 3). Similarly, Canini *et al*. show that CEC and clay content are soil parameters that strongly affect total variance and taxonomic composition of fungal communities [71]. We assume that the importance of CEC and clay content in our correlation analysis results from the direct connection between soil CEC, nutrient-holding capacity and soil clay content [72]. In our study, the C:N ratio had a weak correlation with soil fungal community attributes, which contrasts with a previous study reporting a strong correlation between variation in soil fungal communities and soil C:N ratio [73]. We assume that the effect size of the C:N ratio depends on the origin of the soil samples, which was forest soils in Adamo *et al.* [73] study compared to agricultural soils in this study.

## Conclusion

Soil health, functions and edaphic parameters are strongly linked to agricultural parameters; therefore, soil parameters have been extensively used in soil health assessments in agriculture. The potential linkage we found between agricultural parameters, fungal community heterogeneity and soil parameters could be further investigated as a metric in soil health assessment.

Despite the constraints inherent to a living lab experiment and using ITS2 as a marker for fungal communities, we believe that our broad findings are consistent with field conditions, and this study yielded meaningful and informative patterns, providing robust hypotheses regarding the relationship between soil microbial communities and both agricultural practices and environmental parameters and highlighting the value of on-farm research for generating ecologically relevant insights. However, the correlations identified in this study do not demonstrate causation but instead serve to generate testable hypotheses. Future experimental work will be required to evaluate these hypotheses and establish causal relationships.

Fungal communities were primarily correlated with the geographic location of samples. This finding should be considered in large-scale analyses when the effects of management practices on soil fungal communities are examined. At the provincial scale in Alberta, farming practices introduced shifts in soil fungal community, functional composition, diversity and heterogeneity. We conclude that management practices affected soil fungal communities, and changes in soil fungal communities' attributes were associated with changes in soil physico-chemical conditions, and fungal community heterogeneity was the most responsive attribute to farming practices in Alberta.

This study revealed fungal responses through genomic analysis of agricultural soils in North America at a provincial scale. While soil fungal communities might have similar responsiveness to land use practices at different locations, due to the importance of geographic location in shaping soil fungal community structure and function, we need to be cautious when making recommendations about soil health improvement strategies, and further research is required both in North America and beyond.

## Supplementary material

10.1099/mic.0.001704 Supplementary Material

## Abbreviations

ASVs: amplicon sequence variants
BD: bulk density
BS: base saturation
CEC: cation exchange capacity
EC: electrical conductivity
ITS: internal transcribed spacer
OM: organic matter content
PERMANOVA: permutational multivariate analysis of variance
RDA: redundancy analysis
SQMP: Soil Quality Monitoring Project

## References

[1] Blum WEH (2005). Functions of soil for society and the environment. Rev Environ Sci Biotechnol.

[2] Gregory AS, Ritz K, McGrath SP, Quinton JN, Goulding KWT (2015). A review of the impacts of degradation threats on soil properties in the UK. Soil Use Manag.

[3] Brevik EC, Cerdà A, Mataix-Solera J, Pereg L, Quinton JN (2015). The interdisciplinary nature of SOIL. Soil.

[4] Doran JW, Zeiss MR (2000). Soil health and sustainability: managing the biotic component of soil quality. Appl Soil Ecol.

[5] Coyne MS, Pena-Yewtukhiw EM, Grove JH, Sant’Anna AC, Mata-Padrino D (2022). Soil health – it’s not all biology. Soil Secur.

[6] Cavicchioli R, Ripple WJ, Timmis KN, Azam F, Bakken LR (2019). Scientists’ warning to humanity: microorganisms and climate change. Nat Rev Microbiol.

[7] Frąc M, Jezierska-Tys S, Yaguchi T (2015). Occurrence, detection, and molecular and metabolic characterization of heat-resistant fungi in soils and plants and their risk to human health. Adv Agron.

[8] Sun J, Irzykowski W, Jedryczka M, Han F (2005). Analysis of the genetic structure of *Sclerotinia sclerotiorum* (Lib.) de bary populations from different regions and host plants by random amplified polymorphic DNA markers. J Integr Plant Biol.

[9] Baldrian P (2017). Microbial activity and the dynamics of ecosystem processes in forest soils. Curr Opin Microbiol.

[10] Tunlid A, Floudas D, Op De Beeck M, Wang T, Persson P (2022). Decomposition of soil organic matter by ectomycorrhizal fungi: mechanisms and consequences for organic nitrogen uptake and soil carbon stabilization. Front For Glob Change.

[11] Bearden BN, Petersen L (2000). Influence of arbuscular mycorrhizal fungi on soil structure and aggregate stability of a vertisol. Plant and Soil.

[12] Behie SW, Bidochka MJ (2014). Nutrient transfer in plant-fungal symbioses. Trends Plant Sci.

[13] Gams W, Diederich P, Põldmaa K, Mueller GM, Bills GF, Foster MS (2004). Biodiversity of Fungi.

[14] Den Boogert P, Sneh B, Jabaji-Hare S, Neate S, Dijst G (1996). Rhizoctonia Species: Taxonomy, Molecular Biology, Ecology, Pathology and Disease Control.

[15] Vibha B, Neelam G (2012). Importance of exploration of microbial biodiversity. Int Res J Biol Sci.

[16] Ali I, Yuan P, Ullah S, Iqbal A, Zhao Q (2022). Biochar amendment and nitrogen fertilizer contribute to the changes in soil properties and microbial communities in a paddy field. Front Microbiol.

[17] Schmidt R, Mitchell J, Scow K (2019). Cover cropping and no-till increase diversity and symbiotroph:saprotroph ratios of soil fungal communities. Soil Biol Biochem.

[18] Lian T, Mu Y, Jin J, Ma Q, Cheng Y (2019). Impact of intercropping on the coupling between soil microbial community structure, activity, and nutrient-use efficiencies. PeerJ.

[19] Ma Z, Tanalgo KC, Xu Q, Li W, Wu S (2022). Influence of tea- *Pleurotus ostreatus* intercropping on soil fungal diversity and community structure. Can J Soil Sci.

[20] Hannula SE, Di Lonardo DP, Christensen BT, Crotty FV, Elsen A (2021). Inconsistent effects of agricultural practices on soil fungal communities across 12 European long‐term experiments. Eur J Soil Sci.

[21] Tedersoo L, Bahram M, Põlme S, Kõljalg U, Yorou NS (2014). Global diversity and geography of soil fungi. Science.

[22] Bahram M, Hildebrand F, Forslund SK, Anderson JL, Soudzilovskaia NA (2018). Structure and function of the global topsoil microbiome. Nature.

[23] Labouyrie M, Ballabio C, Romero F, Panagos P, Jones A (2023). Patterns in soil microbial diversity across Europe. Nat Commun.

[24] Aguirre-Monroy AM, Santana-Martinez JC, Lanoil B, MacKenzie MD Drivers of soil bacterial community composition in agricultural systems in alberta and impact of farming practices on bacterial community attributes. Appl Soil Ecol.

[25] Barreiro A, Fox A, Jongen M, Melo J, Musyoki M (2022). Soil bacteria respond to regional edapho-climatic conditions while soil fungi respond to management intensity in grasslands along a European transect. Appl Soil Ecol.

[26] Fox A, Widmer F, Lüscher A (2022). Soil microbial community structures are shaped by agricultural systems revealing little temporal variation. Environ Res.

[27] Cathcart J, Cannon K, Heinz J (2008). Selection and establishment of Alberta agricultural soil quality benchmark sites. Can J Soil Sci.

[28] Norris CE, Bean GM, Cappellazzi SB, Cope M, Greub KLH (2020). Introducing the North American project to evaluate soil health measurements. Agron J.

[29] Carter MR, Gregorich EG (2007). Soil Sampling and Methods of Analysis.

[30] Ihrmark K, Bödeker ITM, Cruz-Martinez K, Friberg H, Kubartova A (2012). New primers to amplify the fungal ITS2 region--evaluation by 454-sequencing of artificial and natural communities. FEMS Microbiol Ecol.

[31] Yang R-H, Su J-H, Shang J-J, Wu Y-Y, Li Y (2018). Evaluation of the ribosomal DNA internal transcribed spacer (ITS), specifically ITS1 and ITS2, for the analysis of fungal diversity by deep sequencing. PLoS One.

[32] Callahan BJ, McMurdie PJ, Rosen MJ, Han AW, Johnson AJA (2016). DADA2: High-resolution sample inference from Illumina amplicon data. Nat Methods.

[33] R Core Team (2023). R: A Language and Environment for Statistical Computing [Internet]. Vienna, Austria: R Foundation for Statistical Computing. https://www.R-project.org/.

[34] Davis NM, Proctor DM, Holmes SP, Relman DA, Callahan BJ (2018). Simple statistical identification and removal of contaminant sequences in marker-gene and metagenomics data. Microbiome.

[35] Schloss PD (2024). Rarefaction is currently the best approach to control for uneven sequencing effort in amplicon sequence analyses. mSphere.

[36] Abarenkov K (2020). UNITE General FASTA Release for Fungi.

[37] McMurdie PJ, Holmes S (2013). phyloseq: an R package for reproducible interactive analysis and graphics of microbiome census data. PLoS One.

[38] Põlme S, Abarenkov K, Henrik Nilsson R, Lindahl BD, Clemmensen KE (2020). FungalTraits: a user-friendly traits database of fungi and fungus-like stramenopiles. Fungal Diversity.

[39] Dixon P (2003). VEGAN, a package of R functions for community ecology. J Veg Sci.

[40] Fox J, Weisberg S (2019). An R Companion to Applied Regression, Third edition.

[41] Ogle D, Ogle MD (2017). Package ‘FSA. Cran Repos.

[42] Harrell Jr FE, Harrell Jr MFE (2019). Package ‘hmisc’. CRAN2018.

[43] Větrovský T, Kohout P, Kopecký M, Machac A, Man M (2019). A meta-analysis of global fungal distribution reveals climate-driven patterns. Nat Commun.

[44] Sommermann L, Geistlinger J, Wibberg D, Deubel A, Zwanzig J (2018). Fungal community profiles in agricultural soils of a long-term field trial under different tillage, fertilization and crop rotation conditions analyzed by high-throughput ITS-amplicon sequencing. PLoS One.

[45] Wang Q, Liang A, Chen X, Zhang S, Zhang Y (2021). The impact of cropping system, tillage and season on shaping soil fungal community in a long-term field trial. Eur J Soil Biol.

[46] Viebahn M, Veenman C, Wernars K, Loon LC, Smit E (2005). Assessment of differences in ascomycete communities in the rhizosphere of field-grown wheat and potato. FEMS Microbiol Ecol.

[47] McKenna TP, Crews TE, Kemp L, Sikes BA (2020). Community structure of soil fungi in a novel perennial crop monoculture, annual agriculture, and native prairie reconstruction. PLoS One.

[48] van der Heijden MGA, Klironomos JN, Ursic M, Moutoglis P, Streitwolf-Engel R (1998). Mycorrhizal fungal diversity determines plant biodiversity, ecosystem variability and productivity. Nature.

[49] Li F, Chen L, Redmile‐Gordon M, Zhang J, Zhang C (2018). *Mortierella elongata's* roles in organic agriculture and crop growth promotion in a mineral soil. Land Degrad Dev.

[50] Ozimek E, Hanaka A (2021). *Mortierella* species as the plant growth-promoting fungi present in the agricultural soils. Agriculture.

[51] Detheridge AP, Brand G, Fychan R, Crotty FV, Sanderson R (2016). The legacy effect of cover crops on soil fungal populations in a cereal rotation. Agric Ecosyst Environ.

[52] Lay C-Y, Bell TH, Hamel C, Harker KN, Mohr R (2018). Canola root-associated microbiomes in the Canadian Prairies. Front Microbiol.

[53] Liu H, Pan F, Han X, Song F, Zhang Z (2020). A comprehensive analysis of the response of the fungal community structure to long-term continuous cropping in three typical upland crops. J Integr Agric.

[54] Zhang H, Luo G, Wang Y, Fei J, Xiangmin R (2023). Crop rotation-driven change in physicochemical properties regulates microbial diversity, dominant components, and community complexity in paddy soils. Agric Ecosyst Environ.

[55] Wang Z, Zhao W, Xi L (2022). Alfalfa cover crops influence the soil fungal community and function in apple orchards in arid desert oases in Northwest China. Sustainability.

[56] Chen H, Ma K, Lu C, Fu Q, Qiu Y (2022). Functional redundancy in soil microbial community based on metagenomics across the globe. Front Microbiol.

[57] Helgason BL, Walley FL, Germida JJ (2010). Long-term no-till management affects microbial biomass but not community composition in Canadian Prairie agroecosytems. Soil Biol Biochem.

[58] Xun W, Yan R, Ren Y, Jin D, Xiong W (2018). Grazing-induced microbiome alterations drive soil organic carbon turnover and productivity in meadow steppe. Microbiome.

[59] Roger-Estrade J, Anger C, Bertrand M, Richard G (2010). Tillage and soil ecology: partners for sustainable agriculture. Soil Tillage Res.

[60] Curaqueo G, Acevedo E, Cornejo P, Seguel A, Rubio R (2010). Tillage effect on soil organic matter, mycorrhizal hyphae and aggregates in a mediterranean agroecosystem. Rev Cienc Suelo Nutr Veg.

[61] Orrù L, Canfora L, Trinchera A, Migliore M, Pennelli B (2021). How tillage and crop rotation change the distribution pattern of fungi. Front Microbiol.

[62] Viterbo A, Horwitz BA (2010). Mycoparasitism. Cell Mol Biol Filamentous Fungi.

[63] Korniłłowicz-Kowalska T, Andruszczak S, Bohacz J, Kraska P, Możejko M (2022). The effect of tillage and no-tillage system on culturable fungal communities in the rhizosphere and soil of two spelt cultivars. Appl Soil Ecol.

[64] Degrune F, Theodorakopoulos N, Dufrêne M, Colinet G, Bodson B (2016). No favorable effect of reduced tillage on microbial community diversity in a silty loam soil (Belgium). Agric Ecosyst Environ.

[65] Helgason BL, Walley FL, Germida JJ (2009). Fungal and bacterial abundance in long‐term no‐till and intensive‐till soils of the Northern Great Plains. Soil Sci Soc Am J.

[66] Li Y, Zhang Q, Cai Y, Yang Q, Chang SX (2020). Minimum tillage and residue retention increase soil microbial population size and diversity: Implications for conservation tillage. Sci Total Environ.

[67] Wang Z, Li T, Wen X, Liu Y, Han J (2017). Fungal communities in rhizosphere soil under conservation tillage shift in response to plant growth. Front Microbiol.

[68] Rose MT, Cavagnaro TR, Scanlan CA, Rose TJ, Vancov T, Sparks DL (2016). Advances in Agronomy.

[69] Nyiraneza J, Thompson BL, geng X, He J, Jiang Y (2017). Changes in soil organic matter over 18 years in Prince Edward Island, Canada. Can J Soil Sci.

[70] Shrestha BM, McConkey BG, Smith WN, Desjardins RL, Campbell CA (2013). Effects of crop rotation, crop type and tillage on soil organic carbon in a semiarid climate. Can J Soil Sci.

[71] Canini F, Geml J, D’Acqui LP, Selbmann L, Onofri S (2020). Exchangeable cations and pH drive diversity and functionality of fungal communities in biological soil crusts from coastal sites of Victoria Land, Antarctica. Fungal Ecol.

[72] Finch HJS, Samuel AM, Lane GPF. (2014). Woodhead publishing series in food science, technology and nutrition. In: Lockhart & Wiseman’s Crop Husbandry Including Grassland (Ninth edition).

[73] Adamo I, Castaño C, Bonet JA, Colinas C, Martínez de Aragón J (2021). Soil physico-chemical properties have a greater effect on soil fungi than host species in Mediterranean pure and mixed pine forests. Soil Biol Biochem.

