# Supplementary material for: Insights into fungal communities in some benchmark agricultural soils of Alberta, Canada
Source: Microbiology (Reading). 2026 May 5;172(5):001704. doi: 10.1099/mic.0.001704 (PMC13142292; doi:10.1099/mic.0.001704)
Supplement: Supplementary Material [file mic-172-01704-s001.pdf]

Table S1. Soil physico-chemical characteristics of sampling sites

Site number	Slope*	NH4 (mg/kg)	NO3 (ppm)	PO4 (ppm)	BS (%)	CEC (meq/100 g)	C:N (%)	pH (CaCl2)	OM (%)	Silt (%)	Clay (%)	EC (dS/m)	BD (kg/L)
586	L	0.6	5	22	100	16.4	12	6.2	5.78	45	29.1	0.27	1.09
586	M	0.5	5	27	87	26.9	14	6.3	7.21	42	31.1	0.27	1
586	U	0.4	5	22	91.2	21.7	12	6.7	5.5	45	29.9	0.2	1.08
592	L	0.5	18	26	100	35.5	10	7.2	11.7	47	26	0.75	0.78
592	M	0.5	30	16	100	30.6	10	6.4	10.3	54	25	0.69	0.69
592	U	0.5	16	29	100	28.2	10	7.3	9.92	49	19.8	0.35	0.81
593	L	0.5	3	14	83.3	20.9	10	5.9	4.71	38	36.9	0.24	1.12
593	M	0.4	6	10	85.1	26.1	10	6.1	6.74	36	38.1	0.1	1.3
593	U	1	5	6	80.9	27.2	11	6	8.7	44	30.8	0.3	1.09
595	L	41	38	34	56.5	24.6	10	5	6.79	49	37	0.48	1.13
595	M	2.5	10	39	53.9	30.6	10	5.2	8.34	43	36.4	0.31	1.22
595	U	1	3	34	67.6	34.3	10	7.7	7.94	35	46	0.28	0.97
599	L	0.6	10	72	81.1	23.8	11	6	9.05	44	41	0.25	1.02
599	M	0.8	4	23	68.3	31.2	11	5.8	9.01	35	37.4	0.22	1.07
599	U	2.2	3	13	69.5	31.5	10	5.7	9.09	20	25.2	0.24	0.99
615	L	3.1	26	16	100	18.8	13	6.8	11.6	46	18	0.5	0.74
615	M	1.7	26	35	100	12.9	13	6.2	9.3	52	14	0.37	0.93
615	U	2.2	9	33	71.6	12	12	6	7.41	54	11.4	0.26	0.86
684	L	3.4	9	66	40.9	15.1	12	5.4	5.74	57	13.1	0.2	1.13
684	M	0.7	5	19	51.3	13.8	12	5	3.65	40	14	0.2	1.26
684	U	0.6	10	31	53.8	14.3	12	5.5	4.8	55	13.1	0.2	1.13
687	L	0.4	9	29	100	16.6	11	6	5.83	33	17	0.29	1.29
687	M	0.3	7	22	84.9	14.6	12	6	4.61	32	10.4	0.21	1.09
687	U	0.7	3	42	70	7.3	10	4.8	1.4	21	5.2	0.1	1.15
688	L	0.4	7	12	69.5	31.5	12	5.8	11	37	21.1	0.1	1.14
688	M	1.1	5	6	71.3	34.5	11	6.2	10.6	47	24.6	0.2	1.08
688	U	0.9	5	7	70.8	32.9	11	6	8.86	42	29.8	0.1	1.24
703	L	0.7	13	20	100	28.8	16	7.1	31.5	23	13.6	0.38	0.6
703	M	0.4	4	6	84	9.5	12	5.7	3.41	29	12.4	0.1	1.26
703	U	0.5	4	7	100	9	11	6	3.42	25	14.9	0.2	1.35
727	L	1.5	7	10	66.7	24.3	12	5.3	14.3	43	21.3	0.2	0.79
727	M	0.4	7	13	65.4	25.4	12	5.5	14.8	33	28.8	0.2	0.91
727	U	0.5	20	40	97.7	20.8	12	6.1	12.7	43	26.6	0.22	0.92
730	L	1.1	3	16	100	26.8	11	7.1	10.7	36	16	0.25	1

730	M	0.4	2	25	54.5	19.8	11	4.8	5.98	26	18	0.08	1.16
730	U	0.7	2	24	100	11	11	5.1	4.5	28	19	0.06	1.22
738	L	2.7	2	9	65.6	15.7	12	6.2	9.79	37	14.4	0.37	1.14
738	M	1	2	12	63	15.1	12	6	8.49	44	17.3	0.2	1.07
738	U	1.3	2	13	72.1	14.7	12	5.9	7.84	38	19	0.1	1.02
740	L	0.4	9	80	100	30.2	11	7.8	14.2	45	15.4	2.72	0.98
740	M	1.6	11	16	54.7	25.2	12	5.8	9.32	36	16	0.1	1.24
740	U	2.3	4	18	60.8	20.9	11	6	5.13	32	20	1.2	1.27
744	L	0.6	9	12	57.7	26.3	12	5.7	11.2	41	18	0.22	0.93
744	M	0.7	8	11	53.3	28.5	12	5.2	9.42	43	20	0.26	0.97
744	U	0.6	10	11	60.7	27.5	12	5.4	6.62	42	23.4	0.31	1.2
746	L	1.3	6	8	66.4	25.9	12	5.9	11.2	39	20	0.1	0.92
746	M	0.5	3	8	75.5	26.9	12	6.5	11.2	41	21.7	0.2	0.99
746	U	0.4	5	10	100	24.8	11	7.5	7.92	34	20	0.45	1
781	L	0.4	15	58	46.3	27.4	11	4.4	10.3	37	33	0.33	0.91
781	M	0.4	9	43	44.7	27.9	12	4.4	8.73	32	31.3	0.25	0.97
781	U	1.5	7	42	52.6	25.1	11	4.4	5.87	33	28.7	0.26	1.08
791	L	0.6	5	25	67.7	19.1	11	5.8	5.99	41	21.7	0.24	0.95
791	M	0.4	6	28	100	24.8	12	6.9	5.22	39	20	0.74	1.14
791	U	0.5	4	25	72	19.3	11	5.7	3.61	37	18	0.2	1.11
793	L	0.3	2	9	100	14	12	6.7	5.1	35	14.7	0.08	1.39
793	M	0.4	2	6	100	17.3	10	7.4	4.7	38	16	0.1	1.53
793	U	0.5	2	15	100	28.6	12	7.6	4.68	38	19	0.22	1.23
800	L	0.5	2	25	99.5	18.9	11	5.5	6.6	40	40	0.08	1.23
800	M	0.7	3	33	100	15.4	12	5.6	7.22	38	37.1	0.1	1.14
800	U	1	3	28	63.6	25.8	12	5.6	7.77	37	37	0.09	1.15
802	L	0.5	6	19	74.6	20.5	11	6	5.56	37	26.4	0.2	0.93
802	M	0.6	5	28	59.2	22.8	12	6.3	6.26	46	21.2	0.21	1.11
802	U	0.4	3	11	100	28.9	10	7.8	4.11	44	24.7	0.26	1.09
804	L	0.3	4	21	60.6	16.3	11	5.4	5.08	41	19	0.26	1.03
804	M	0.3	4	17	57.3	13.3	8.8	5.2	5.02	38	14	0.1	1.13
804	U	0.3	2	8	100	12	7.4	5.8	2.73	31	14	0.1	1.19

809	L	0.4	7	13	88.2	11	11	5.9	4.26	47	30.3	0.07	1.11
809	M	0.3	6	15	100	8.2	11	5.8	2.66	37	18.9	0.06	1.27
809	U	0.3	4	6	100	16.5	9.9	7.3	2.6	32	24	0.1	1.27
812	L	0.4	6	12	100	30.6	10	7.7	3.07	34	26.3	0.28	1.19
812	M	0.4	10	23	100	30.1	10	8.1	3.11	41	21.1	0.47	1.1
812	U	0.4	5	12	11	30.3	10	8.1	2.57	35	22.8	0.28	1.23
815	L	0.4	44	46	77	12.6	10	5.3	5.19	45	17.6	0.42	1.35
815	M	0.3	5	11	100	17.1	10	7.2	2.65	34	9.6	0.2	1.25
815	U	0.3	6	14	100	22.9	12	7.2	4.53	37	16.5	0.2	1.4
823	L	72.7	6	30	100	13.4	9.5	7.6	3.16	42	14.9	0.42	1.21
823	M	20.2	3	28	100	14.9	9.3	7.8	2.9	46	20.7	0.5	1.2
823	U	28	7	80	100	19.4	9.3	7.7	3.72	49	22.3	0.41	1.19

***L: Lower M: Middle U: Upper**

Table S2. Summary of survey responses regarding agricultural practices at each site. Only questions relevant to this study are included in the table.

Site number	Ecoregion*	Tillage**	Crop	Crop details	Herbicide	Herbicide compound
586	PL	High	Wheat	Barley	Herbicide	Glyphosate
592	PL	Low	Perennial	None	None	None
593	PL	Low	Wheat	Wheat	Herbicide	Glyphosate
595	PL	Low	Wheat	Wheat	None	None
599	PL	Low	Wheat	Wheat	Herbicide	Glyphosate
615	MB	Low	Perennial	Livestock	Herbicide	Glyphosate
684	BT	Zero	Canola	Canola	Herbicide	Glyphosate
687	BT	Zero	Wheat	Wheat	Herbicide	Glyphosate
688	BT	Zero	Canola	Canola	Herbicide	None glyphosate
703	BT	Low	Wheat	Wheat	Herbicide	Glyphosate
727	AP	Low	Wheat	Barley	Herbicide	Glyphosate
730	AP	Low	Perennial	Livestock	None	None
738	AP	Low	Perennial	Hay	None	None
740	AP	Low	Canola	Canola	None	None
744	AP	High	Wheat	Wheat	Herbicide	None glyphosate
746	AP	Zero	Perennial	Forage	None	None
781	MM	Zero	Canola	Canola	Herbicide	Glyphosate
791	MM	Zero	Canola	Canola	Herbicide	Glyphosate
793	MM	Zero	Wheat	Wheat	Herbicide	Glyphosate
800	MG	High	Wheat	Barley	Herbicide	Glyphosate
802	FG	Zero	Wheat	Wheat	Herbicide	Glyphosate
804	MG	Zero	Perennial	Fallow	None	None
809	MG	Low	Wheat	Durum	Herbicide	Glyphosate
812	MG	Zero	Other	Alfalfa seed	None	None
815	MG	Zero	Wheat	Wheat	Herbicide	None glyphosate
823	MG	Low	Other	Sugar beets	Herbicide	None glyphosate

***PL: Peace Lowland MB: Mid-Boreal Uplands BT: Boreal Transition AP: Aspen Parklands MM: Moist Mixed Grasslands MG: Mixed Grasslands FG: Fescue Grasslands.**

****Tillage=Number of passes, if ≥2 High, if =1 Low, and if=0 Zero.**

Table S3. Sample grouping based on farming practices and ecoregions

**Crop**	**Canola**	**Perennial (forage, fallow, livestock)**	**Grains (barley, durum, wheat)**
Farm	5	4	13
Ecoregion	AP, BT, MM	AP, MB, MG	AP, BT, FG, MG, MM, PL
Tillage	Low, Zero	Low, Zero	High, Low, Zero
Herbicide	Herbicide, No herbicide	Herbicide, No herbicide	Herbicide, No herbicide
**Tillage**	**High**	**Low**	**Zero**
Farm	3	12	11
Ecoregion	AP, MG, PL	AP, BT, MB, MG, PL	AP, BT, FG, MG, MM
Crop	Grains	Canola, Perennial, Grains	Canola, Perennial, Grains
Herbicide	Herbicide	Herbicide, No herbicide	Herbicide, No herbicide
**Herbicide**	**Herbicide (glyphosate, non-glyphosate)**	**No herbicide**	
Farm	18	8	
Ecoregion	AP, BT, FG, MB, MG, MM, PL	AP, MG, PL	
Crop	Canola, Perennial, Grains	Canola, Perennial, Grains	
Tillage	High, Low, Zero	Low, Zero	

Table S4. Most abundant fungal functional groups obtained from FungalTraits database evaluated in this study with number of fungal genera associated to that genus.

Fungal trait	Number of genera	Number of ASVs	Examples of genera (number of ASVs)
Soil saprotroph	104	1341	Pseudogymnoascus (52), Mortierella (265), Solicoccozyma (42)
Litter saprotroph	104	700	Chaetomium (33), Tetracladium (91), Cladophialophora (25)
Mycoparasite	22	197	Fusicolla (16), Trichoderma (45), Sporobolomyces (9)
Wood saprotroph	93	520	Humicola (11), Clonostachys (5), Chrysosporium (42)
Unspecified saprotroph	54	396	Penicillium (42), Coniochaeta (93), Filobasidium (14)
Dung saprotroph	21	341	Thelebolus (12), Podospora (57), Kernia (15)
Animal parasite	32	209	Exophiala (24), Metarhizium (29), Phialemonium (11)

Table S5. Indicator species analysis in each agricultural parameter, showing only top 10 indicator species with the highest chance of appearance.

Tillage	ASV number	Statistical support	p-value		Crop	ASV number	Statistical support	p-value		Herbicide	ASV number	Statistical support	p-value
High	ASV4	0.637	0.0001		Other	ASV117	0.926	0.0001		None	ASV109	0.347	0.0001
High	ASV13	0.628	0.0001		Other	ASV54	0.91	0.0001		None	ASV417	0.333	0.0008
High	ASV1033	0.596	0.0001		Other	ASV141	0.878	0.0001		Herbicide	ASV163	0.321	0.0031
High	ASV4338	0.59	0.0001		Other	ASV161	0.861	0.0001		Herbicide	ASV114	0.32	0.0014
High	ASV164	0.55	0.0001		Other	ASV377	0.857	0.0001		None	ASV410	0.32	0.002
High	ASV114	0.55	0.0001		Other	ASV37	0.817	0.0002		Herbicide	ASV72	0.319	0.0021
High	ASV38	0.543	0.0001		Other	ASV40	0.775	0.0001		None	ASV288	0.319	0.0005
High	ASV340	0.528	0.0004		Other	ASV59	0.75	0.0001		None	ASV449	0.316	0.0001
High	ASV1095	0.519	0.0002		Other	ASV338	0.75	0.0001		None	ASV756	0.315	0.0013
High	ASV237	0.519	0.0003		Other	ASV2398	0.736	0.0001		None	ASV1063	0.31	0.0017
High	ASV112	0.51	0.0006		Other	ASV2947	0.724	0.0001		Herbicide	ASV258	0.299	0.0033
High	ASV729	0.509	0.0006		Other	ASV109	0.719	0.0001		None	ASV727	0.294	0.0022
High	ASV1417	0.509	0.0004		Other	ASV916	0.699	0.0001		Herbicide	ASV14	0.293	0.0067
High	ASV4066	0.5	0.0012		Other	ASV11	0.697	0.0001		None	ASV217	0.293	0.0039
High	ASV1206	0.498	0.0012		Other	ASV60	0.685	0.0002		Herbicide	ASV343	0.292	0.0111
High	ASV505	0.494	0.0009		Other	ASV667	0.672	0.0001		None	ASV78	0.291	0.0017
High	ASV1756	0.482	0.0005		Other	ASV247	0.657	0.0001		Herbicide	ASV103	0.29	0.0122
High	ASV2356	0.482	0.0009		Other	ASV2117	0.655	0.0003		Herbicide	ASV577	0.289	0.006
High	ASV1504	0.48	0.0007		Other	ASV2708	0.655	0.0004		Herbicide	ASV12	0.286	0.0132
High	ASV749	0.475	0.0003		Other	ASV2799	0.655	0.0002		Herbicide	ASV889	0.284	0.0252

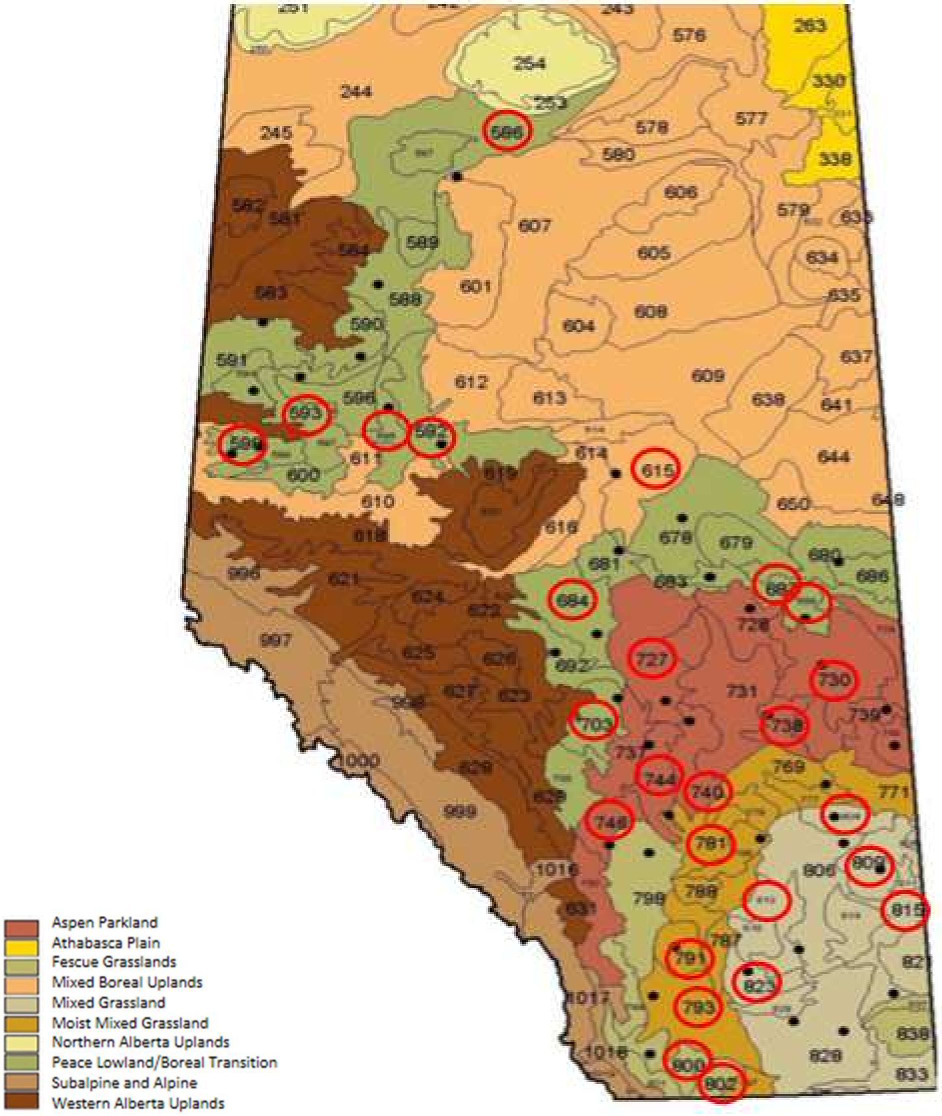

Figure S1. Locations of Soil Quality Monitoring Project sites from the original study (Cathcart, et al., 2008). Shading shows different ecoregions across Alberta. Numbers indicate the eco-district of the site and colors indicate different ecoregions (Cathcart et al., 2008). Red circles show the sites assessed in this study.

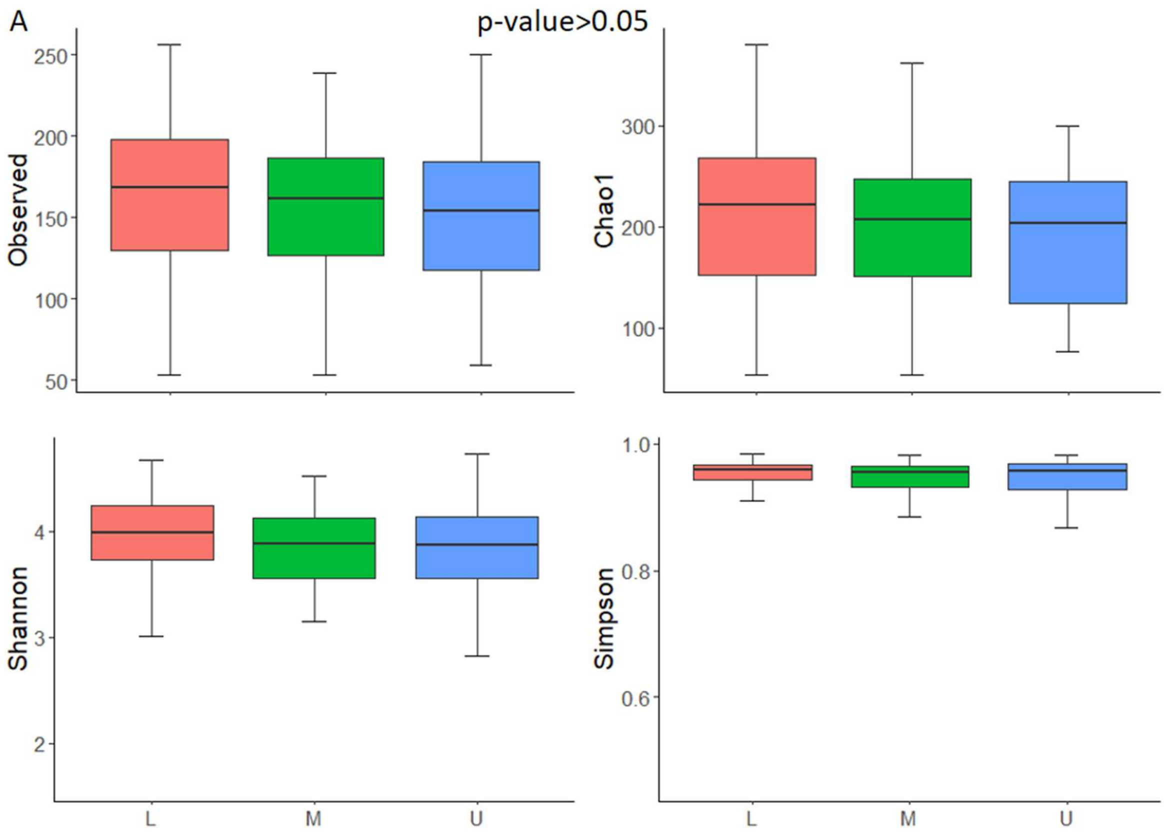
A
p-value>0.05
Observed
250
200
150
100
50
Chao1
300
200
100
Shannon
4
3
2
Simpson
1.0
0.8
0.6
L
M
U
L
M
U

B

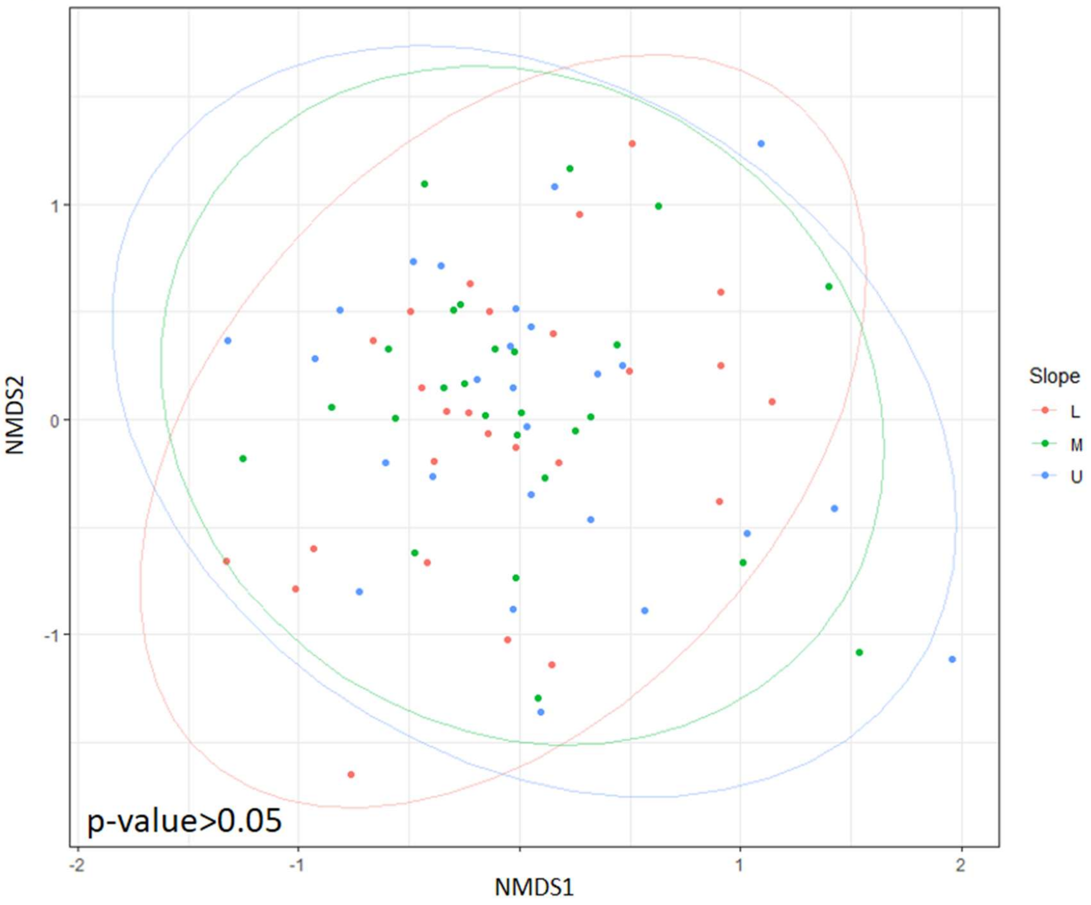

C

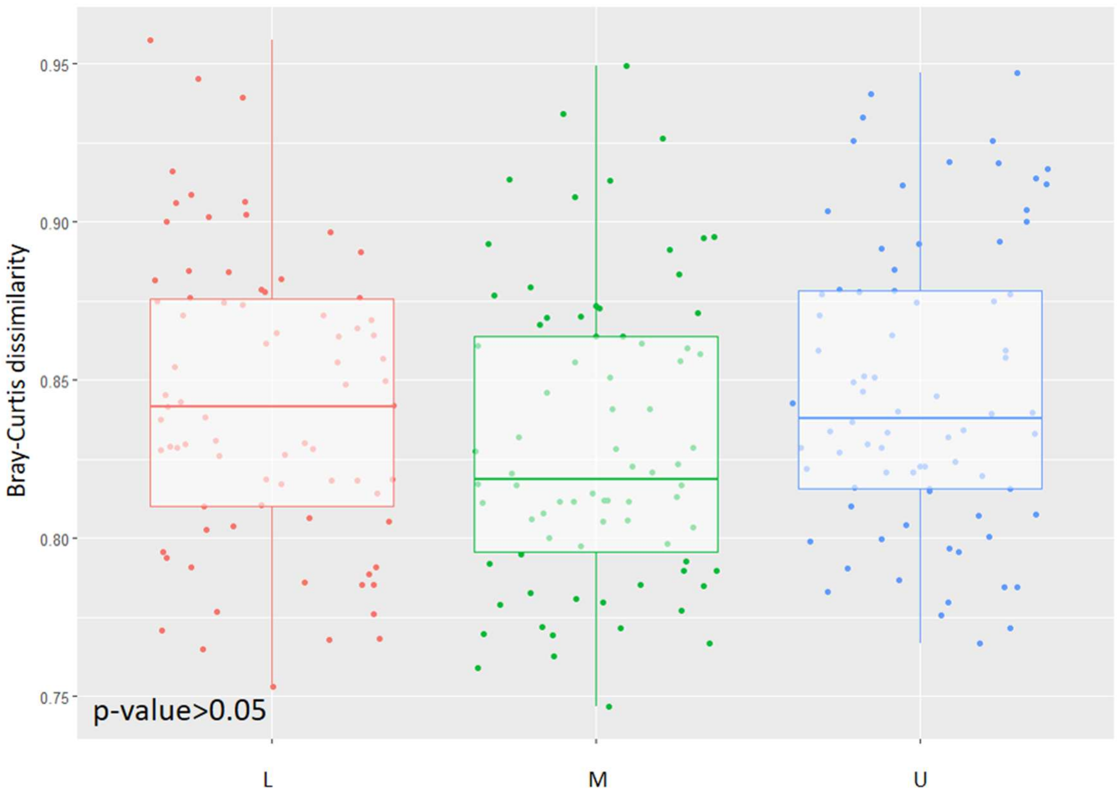

Figure S2. Comparison of alpha-diversity metrics (Observed, Chao1, Shannon Index, and Simpson Index) (A), species composition by NMDS ordination of Bray-Curtis dissimilarity (B), and pairwise Bray-Curtis dissimilarity (a measure of community heterogeneity) (C) between slope positions along a catena from benchmark sites.

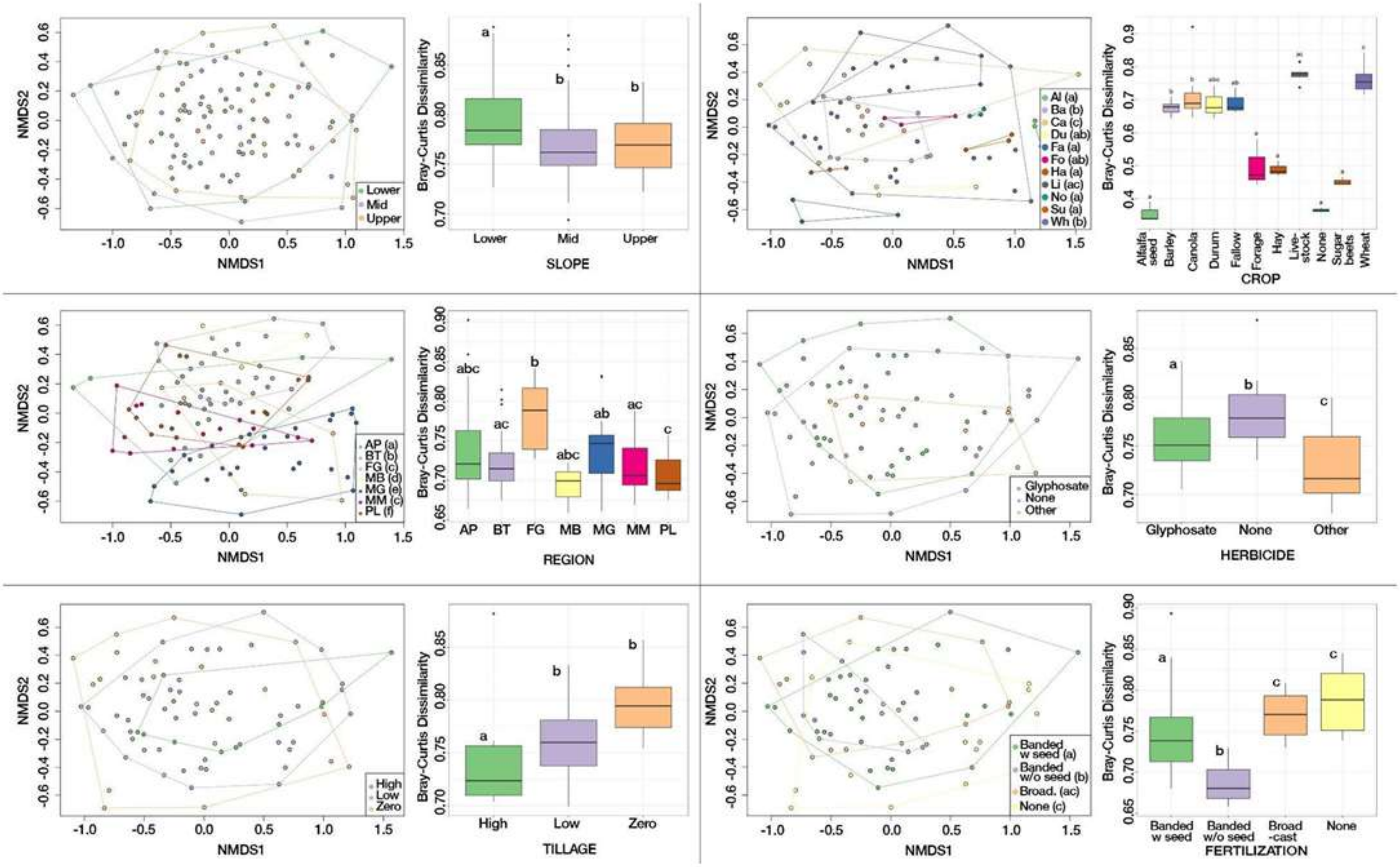

Figure S3. Non-metric multidimensional scaling ordination (NMDS) and corresponding Bray-Curtis dissimilarity of bacterial community composition at the ASV level, clustered according to each of the six evaluated factors: slope position, ecoregion, tillage intensity, crop types, herbicide use, and fertilization methods. Lower case letters in the ordination legend and the boxplots indicate significant differences ($p < 0.05$) according to pairwise Adonis and pairwise Wilcoxon Rank tests respectively. Ordination stress for NMDS clustered by slope and ecoregion = 0.171282. Ordination stress for NMDS clustered by the different agricultural practices = 0.154606. AP: Aspen Parkland; BT: Boreal transition; FG: Fescue Grassland; MB: Mixed Boreal; MG: Mixed grassland; MM: Mixed Moist Grassland; PL: Peace Lowland.
